# On the Undiminished Mortality from Puerperal Fever in England and Wales

**Published:** 1897-03-06

**Authors:** Charles J. Cullingworth

**Affiliations:** President of the Obstetrical Society, Obstetric Physician to and Lecturer on Diseases of Women at St. Thomas's Hospital.


### ON THE UNDIMINISHED MORTALITY FROM PUERPERAL FEVER IN ENGLAND AND WALES.

By Charles J. Cullingworth, M.D., F.R.C.P., President of the Obstetrical Society, Obstetric Physician to and Lecturer on Diseases of Women at St. Thomas's Hospital.*

After expressing his high appreciation of the honour conferred upon him in appointing him president of the society, Dr. Cullingworth proceeded to say that, on the principle that more is often learned from a record of failure than from a story of success, he proposed to draw the attention of his profession to the death-rate from puerperal fever in this country, and to the humiliating fact that, notwithstanding the introduction of antiseptics, and the almost complete banishment of the disease from our lying-in hospitals, and the general advance in our obstetric knowledge, the death-rate had not only not diminished, but in some districts had actually advanced during the past few years. Others had already drawn attention to this, but he thought the profession had hardly as yet realised the enormous importance and significance of the facts, and had not laid to heart the lessons to be learnt from them or the urgent necessity which existed for making a radical alteration in our present midwifery practice.

After showing by tables of mortality how considerably the deaths from puerperal fever had increased since 1847, when records on the subject began to be kept by the Registrar-General, he admitted that the statistics were to some extent affected by the fact that of late years inquiries had been instituted in regard to cases returned as peritonitis, septicæmia, or pyæmia, in women of child-bearing age, with the result that a certain number of these had been transferred to the heading of puerperal fever. But after all due allowances had been made, we were still face to face with the fact that there had been an increase, and that the year 1893 was conspicuous for a puerperal fever mortality that had only once been exceeded since 1849. He then drew attention to the geographical distribution of the disease, especially referring to its great prevalence in some of the wealthier parishes in London. In London the total death-rate from child-birth had considerably diminished, but the diminution was due, not to any appreciable lowering of the mortality from puerperal fever, but to a lessened mortality from the accidents of child-birth. In the provinces, on the other hand, the total death-rate from child-birth remained practically unaltered, the increased mortality from puerperal fever having balanced and neutralised any such diminution as had occurred from the accidents of child-birth. Between 1885 and 1894, Lancashire, Cheshire, and North and South Wales held an unenviable pre-eminence in the black list.

Quoting Dr. Williams, medical officer of health for the county of Glamorganshire, he drew a contrast between three districts in that county, one of which had a puerperal mortality exactly corresponding with that of the whole of England and Wales, while the others had a mortality more than twice as high. In Cardiff "the sanitary condition is satisfactory, and confinements are attended generally by the medical men." In

* Inaugural Presidential Address delivered before the Obstetrical Society, March 3rd, 1897.

the Pontypridd district the puerperal mortality was excessive (4·1). Its sanitary condition was generally unsatisfactory, and most of the confinements were attended by "untrained midwives." In the Merthyr Tydvil district the death-rate from puerperal fever was higher than in any other district in Wales, viz., 4·7 per 1,000 births. The greater number of confinements were attended by midwives, of whom only a few, if any, were trained.

"From my experience in Glamorganshire," said Dr. Williams, "I can prove, without a doubt, that the midwives often spread puerperal fever broadcast, and are often not interfered with." And he went on to say, "The conditions of life consequent upon the physical features of the country, especially in North Wales, often make it impossible to obtain skilled assistance in time, and if any is to be obtained it must be from untrained midwives." Even these were not always available, and in such districts friends and neighbours were often compelled to give the help that was needed. Evidence was brought forward to show that much the same state of affairs was prevalent in Lancashire; an excessive mortality from puerperal fever being coincident with a general prevalence of the custom of employing midwives.

It must not, however, be supposed, said Dr. Cullingworth, that I am going to lay the entire blame for the present deplorable state of affairs upon the midwives. The evidence is incontestible that they are responsible for much of it, but on this head I have probably for the present said enough; I desire now to bring the inquiry nearer home.

Puerperal fever still claims its victims not only in thinly-peopled country districts, but in places densely populated, where, whatever the causes may be, the difficulty of obtaining medical assistance cannot be one of them. Although puerperal fever has practically become a thing of the past in our lying-in hospitals, it is impossible to ignore the fact that there has not been a corresponding diminution of mortality from this disease in ordinary private practice. Either the profession is not convinced of the possibility of stamping out puerperal fever by the methods that have been proposed, or it has failed to carry out those methods with the thoroughness that can alone ensure success.

I have often asked myself, said Dr. Cullingworth, do we teachers sufficiently impress upon the minds of our students the infinite importance of this subject? If we do not, is it to be wondered at that so many men should discharge their consciences in the matter of antiseptics by pouring a few drops of carbolic acid or a drachm of tincture of iodine into the water in which they wash their hands, or by simply bidding the nurse to administer a similarly-prepared solution as a douche? The methods at present employed by many who would assure one that they had used "every antiseptic precaution" are often so crude and imperfect as to be a ludicrous travesty of genuine antiseptic midwifery. It is clear that something is wrong.

A good deal has recently been written on the superiority of asepsis as compared with antisepsis, and it has become the fashion in some quarters to speak of antiseptics as though they had had their day, and to maintain that a condition of asepsis can be attained by cleanliness alone. It is well, however, to bear in mind that the triumphs of our lying-in hospitals have all been attained by the scrupulous use of antiseptics, and that sterilisation, which is the essential element in aseptic surgery, is impossible in midwifery practice. As a matter of experience it is constantly observed that those who are most diligent in the use of antiseptics are also the most diligent in carrying out the details of elementary cleanliness.

On an occasion like this the temptation was strong upon me to choose some newer topic than the somewhat trite one that I have dealt with to-night. But the more I reflected on the matter the more it was borne in upon me that it was my duty again to raise my voice on behalf of the mothers of this country. If anything I have said should have the effect of stirring the minds of my professional brethren on this subject, and of diminishing, to ever so slight an extent, the present terrible death-rate from a disease which is now proved to be almost wholly preventable, I shall feel that I have achieved something worthy of the occasion, and have done something towards justifying your choice of a president.

The address was illustrated by various maps and diagrams, and, in regard to London, it was a startling thing to find how very largely puerperal fever prevailed in certain parishes, such as Hampstead, Wandsworth, and Lewisham, in which the general mortality was very low—in places specially selected as homes for the wealthy, districts in which medical men and nurses of the highest class abound—and one could not but feel that some grave lapse in the conduct of antisepsis must be very common in general midwifery practice, if so many women could die, where the surroundings otherwise were so good, from a disease like puerperal fever; for by the careful use of these antiseptic methods, that same disease has been banished from institutions situated in most crowded parts of London, and used by people whose sanitary antecedents are as unfavourable as they possibly could be.

